# Psycheutopia: an innovative educational program to enhance mental health literacy among medical students

**DOI:** 10.3389/fpsyt.2025.1538476

**Published:** 2025-03-27

**Authors:** Zahra Jabari, Mohammad Eslami, Amir Hossein Jalali Nadoushan, Salime Goharinezhad, MohammadJavad Tavallaei, Elaheh Khodadoust, Seyed Mohammad Hossein Mahmoodi

**Affiliations:** ^1^ Faculty of Medicine, Iran University of Medical Sciences, Tehran, Iran; ^2^ Mental Health Research Center, Psychosocial Health Research Institute (PHRI), Department of Psychiatry, School of Medicine, Iran University of Medical Sciences, Tehran, Iran; ^3^ School of Health, Wellbeing and Social Care, The Open University, Milton Keynes, United Kingdom; ^4^ Health Policy Research Center, Institute of Health, Shiraz University of Medical Sciences, Shiraz, Iran

**Keywords:** mental health literacy, mental health education, mental health intervention, medical students, subjective well-being, stress management, depression, suicide

## Abstract

**Introduction:**

Medical students face a high prevalence of mental disorders, such as depression, alongside inadequate mental health literacy (MHL). This study investigates the feasibility, and efficacy of an interactive, gamified educational program in improving participants’ MHL.

**Method:**

Thirty-two Iranian medical students participated in a semi-experimental pretest-post-test study. An 18-hour online program, delivered using the flipped classroom method, focused on mental health first aid, stigma, stress management, and subjective well-being (SWB) skills. Participants were evaluated before, after, and four months post-program using the Mental Health Literacy Scale (MHLS), Mental Health Promoting Knowledge (MHPK), and World Health Organization-5 Well-being Index (WHO-5).

**Results:**

Participants' mean age was 22.78 ( ± 4.54). Post-test results showed significant improvement in MHLS and MHPK scores (P-values< 0.05), which were sustained at the four-month follow-up (P-values>0.01). SWB improved in the post-test evaluation (P-value= 0.058).

**Conclusion:**

The educational program effectively improved medical undergraduates’ MHL and SWB. Given the rising concerns about medical students’ mental health, implementing such curricula appears promising for long-term benefits.

## 1 Introduction

The World Health Organization (WHO) defines health as a state of complete physical, mental, and social well-being, not merely the absence of disease or infirmity (1). Despite this definition, which emphasizes mental health as a key domain, mental health disorders account for around 14% of the global disease burden and eight million deaths annually (2, 3). Each year, about 800,000 people commit suicide, with a notable number of them being young individuals aged 15-24 (4).

The prevalence of mental health issues among medical science students is significantly higher than in the general population, with over half of those studying medicine experiencing psychological tensions (5–8). Furthermore, our previous investigation revealed low scores among university students. Additionally, there was a high rate of individuals screened positive for clinical depression (9). A meta-analysis found that approximately 28% of 17,560 medical students experience depression (10). This issue is also emphasized in many studies worldwide (11–14). Another meta-analysis showed that the prevalence of depression among medical students in the Middle East is around 31.8 %, which is more than the prevalence of depression in medical students in North America (30.3%), Asia (30.1%), South America (26.8%), and Europe (20%) (7).

In Iran, and specifically among medical students, the prevalence of depression and suicide ideation has become a rising concern (10, 15–18). According to the Iranian Psychiatric Association, there have been 16 cases of suicide by resident doctors in just nine months (19).

These estimates suggest that mental health disorders among medical students contribute to a significant public health challenge, necessitating urgent interventions to address this burden (20, 21). Improving mental health literacy (MHL), defined as 'knowledge and beliefs about mental disorders that aid in their recognition, management, or prevention,' can empower communities to pursue better mental health (22, 23). This definition is complemented by positive mental health literacy, which refers to a person’s knowledge about improving and preserving good mental health (23).

The WHO's comprehensive mental health action plan identifies public mental health awareness as one of the initial strategies in mental health prevention, with higher MHL associated with better mental health outcomes (24). In Iran, a cross-sectional study revealed a significant lack of MHL among medical students (25, 26). MHL is considered a modifiable factor (27, 28), and empowering students with resilience and mental health-related skills has been identified as a student support strategy (29). Developing preventive and educational interventions for this at-risk population is suggested, with improving MHL showing cost-effective and promising results (27, 30, 31). A systematic review of mental health first aid training trials revealed that improvement in different aspects of MHL is achievable, but long-term improvements are still a challenge (32).

While training programs are recommended to improve MHL, interactive teaching methods are necessary to create effective and practical learning. Studies suggest that methods such as gamification and scenario-based learning enhance educational outcomes by actively engaging participants and fostering critical thinking. These methods have shown promise in improving mental health literacy by creating immersive and memorable learning experiences (33–36). A systematic review by Mohammadi et al. (2020) highlighted the effectiveness of interactive methods, including role-playing, games, and multimedia approaches, in mental health literacy interventions among adolescents (37). However, their application in medical students remains underexplored. Recognizing the importance of improving mental health literacy (MHL) among medical students, our team developed and implemented an interactive, didactic program called *Psycheutopia* for a group of Iranian medical students. This initiative aimed to enhance their MHL as a critical step toward fostering their psychological well-being. In essence, our study had two primary objectives: first, to design a program tailored to improve MHL among medical students, and second, to evaluate its effectiveness.We hypothesize that Psycheutopia will significantly improve mental health literacy (MHL) and subjective well-being (SWB) among medical students.

## 2 Method

**Figure 1:**
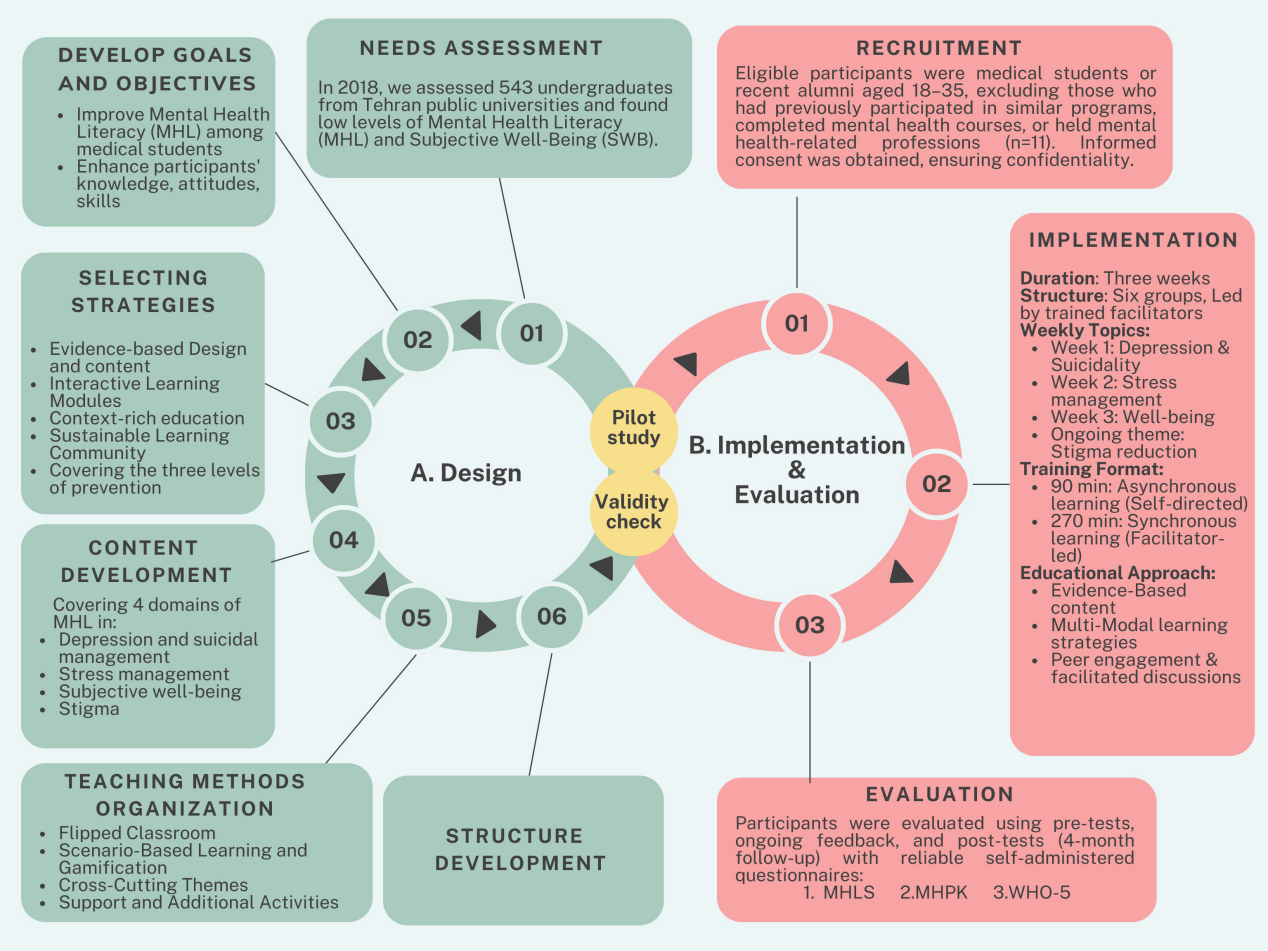
Overview of the Study Phases: The study was conducted in multiple phases, including the design, implementation & evaluation as the main phases. The figure illustrates the progression of these phases, **(A)** starting with program design and refinement, followed by validation and pilot testing to ensure feasibility. **(B)** The final phases involved program implementation and its subsequent evaluation to assess outcomes and effectiveness.

In this study, we designed a curriculum and evaluated its efficacy in improving MHL. The study method contains two phases: A. Design and module development and B. implementation & evaluation. Each phase is explained below (**Figure 1**).

### 2.1 Design

This curriculum was developed based on Harden's ten questions for curriculum planning as a guiding framework (38). After conducting a needs assessment study, we established the goals and objectives for this curriculum and selected relevant content and effective teaching strategies. The initial version of the program was piloted as a three-day in-person event in February 2020 and a sample size of 20 to 30 participants was considered sufficient for the pilot.

In response to the emerging COVID-19 pandemic, this program was subsequently adapted to an online format.

#### 2.1.1 Needs assessment

In 2018, our team conducted a descriptive cross-sectional study to measure the levels of MHL and SWB among the undergraduates of Tehran public universities. MHL qualities were measured in 543 students and the results revealed insufficient MHL and WB among the participants (26).

#### 2.1.2 Objectives

Our primary objective was to address the issue of inadequate MHL among students. As the initial step to improve MHL and Subjective Well-Being (SWB) among them, we concentrated on medical students. Despite mental disorder diagnosis and treatment being part of their curriculum, our needs assessment revealed their MHL levels were no different from those of other students.

In response to the needs assessment, we designed *Psycheutopia* as a comprehensive program with these objectives:

- Develop an effective educational program to enhance mental health literacy among medical students.
- Cater to the diverse educational needs of participants by covering topics across different levels of mental health prevention: illness first aid, primary prevention, and promotion.
- Improve participants’ knowledge, attitude, and, particularly, skills.
- Establish a learning community where educators, organizers, and participants can collectively learn, apply, and disseminate the acquired knowledge in the future.

#### 2.1.3 Strategies

To accomplish these ambitious objectives, we devised five key strategies:

- Evidence-based Design and content: Utilize exclusively evidence-based learning methods and materials.
- Interactive Learning Modules: Create a genuinely interactive educational environment that is also enjoyable to boost learners’ motivation and active participation throughout an online program.
- Context-rich education: Offer an education that is rich in context, making the topics applicable to the participants' daily lives. This approach helps translate knowledge into practice and fosters the development of higher cognitive skills in line with Bloom’s taxonomy.
- Covering the three levels of prevention: prepare the program in a way not only to reduce the risk of being involved by mental disorders but also to be capable of facing it and overcoming it during our lives.
- Sustainable Learning Community: Foster an ongoing learning community where participants can continuously share knowledge and best practices.

#### 2.1.4 Educational content

To pursue our objectives, the curriculum was constructed based on the four domains of MHL (22):

1. Understanding how to obtain and maintain good mental health.
2. Understanding mental disorders and treatment.
3. Decreasing stigma against mental illness.
4. Enhancing help-seeking efficacy.

A part of our curriculum focused on early detection and immediate support for mentally distressed peers, emphasizing self-care strategies during crises. This section specifically addressed depression and suicidality, the most common mental health challenges, and was adapted from the evidence-based Mental Health First Aid (MHFA) action plan. MHFA has demonstrated effectiveness in enhancing mental health literacy, reducing stigma, and fostering supportive behaviors (32, 36, 39, 40).

Secondly, medical students are dealing with daily psychological stress that makes them vulnerable to burnout, depression, and anxiety disorders. Learning stress management skills and fostering resilience were included in the program’s content to help students prevent mental disorders and promote their SWB and productivity (41).

Moreover, based on the World Health Organization’s (WHO) definition of health, being healthy is not just the absence of disease but a satisfactory status of well-being (1). Positive mental health literacy, which refers to a person’s knowledge about enhancing and preserving good mental health, was also addressed in this curriculum (42, 43).

Finally, stigma was approached independently in this program. Participants were challenged to think about different types of stigmas in various contexts and their roles in destigmatizing mental disorders and facilitating help-seeking behaviors.

**Figure 2:**
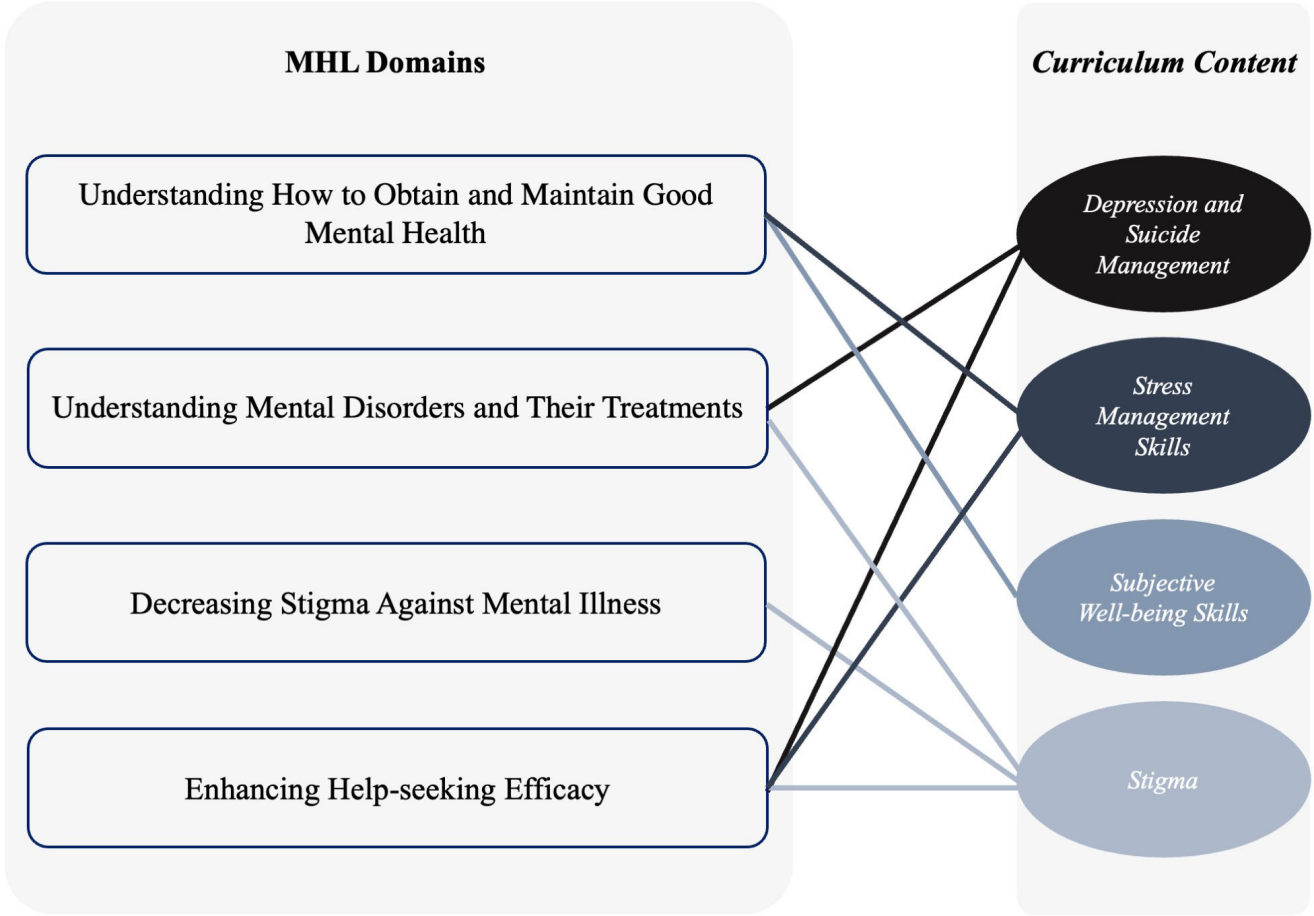
Domains of mental health literacy: the figure illustrates the four core domains of mental health literacy addressed in this study, which formed the basis for the program, 's content development.

Therefore, our content concentrated on four topics to address all domains of MHL (**Figure 2**). The content validity was verified by experts who reviewed and critically revised all created learning materials. Initially, in three sessions involving one psychiatry resident and three general medical students, the findings of previous studies on students' mental health literacy (MHL) were reviewed. This included a needs assessment previously conducted by our team and global studies aimed at improving MHL, such as those based on Mental Health First Aid (MHFA). Educational topics were selected with consideration of the cultural and linguistic context of the target population. In the next step, essential scientific content for these topics was gathered from public education resources. Subsequently, using scenario-based methods, games, and other interactive techniques, the format for delivering this content was developed. The content and its delivery format were then evaluated by two experts—a psychiatry resident and a psychiatry professor—who provided necessary revisions. This process was repeated until content validity and face validity were confirmed by the experts. Finally, to complete the assessment of face validity, four individuals from the target population also reviewed the materials.

##### 2.1.4.1 Teaching methods

Aiming to create an online, interactive, and context-rich learning environment to foster life skills, and considering our objectives, we used various educational methods, including flipped classroom, scenario-based learning, gamification, self-reflective learning, and cross-cutting themes.

###### 2.1.4.1.1 Flipped classroom

The program included 4.5 hours of asynchronous and 13.5 hours of synchronous sessions over three weeks. Asynchronous materials like educational videos were provided online, with support from facilitators. Weekly synchronous sessions involved missions and group activities to reinforce learning (43, 44).

###### 2.1.4.1.2 Scenario-based learning and gamification

Activities were designed around an engaging scenario with participants playing key roles in providing a context-rich environment. Gamification strategies such as storylines, instant feedback, and leaderboards were used to increase motivation and engagement (40).

###### 2.1.4.1.3 Cross-cutting themes

The theme of stigma was integrated throughout the program with the aim of more in-depth training to change attitudes.

###### 2.1.4.1.4 Support and additional activities

Participants received a mailed educational package to bridge the gap between online and in-person experiences. Additional support included an anonymous chat box for personal questions and weekly Q&A sessions with psychiatric residents. Emergency hotlines were also provided for ongoing support.

Teaching methods were adjusted to suit specific educational objectives, with different methods used for different topics, such as scenario-based learning for mental health first aid and self-reflective learning for subjective well-being. Examples of these educational activities are summarized in **Table 1**.

**Table 1:** Examples of activities employed in the program.

Topic	Activity’s Title	Educational Method					S or A	Explanation of The Activity
		SBL	SGD	GBL	SRL	RP		
Suicide Prevention	Confidential Data	✓		✓			A	An asynchronized activity to improve the skills of recognizing warning signs of suicide. Students listen to five audio tapes introducing five characters struggling with suicidal ideas, and examine the characters’ profiles and try to find the specific signs which represent suicidal ideation based on what they have learned in an educational video.
	Errors and suggestions	✓	✓	✓			S	Students listen and read a couple of voices about people describing their conversation with a suicidal person. Their task is to identify the speaker’s errors and suggest a better response through a group discussion. They can choose from the provided *‘Error Cards’* and *‘Suggestion Cards’.*
Depression Management	Darya’s Letter	✓		✓			A	In this asynchronous activity, students will read a letter written by a character named Darya, provided through the program's scenario. Their task is to identify signs and symptoms of depression within the letter.
	Darya’s Footprint					✓	S	Two student volunteers will role-play a scenario where one acts as a person experiencing depressive symptoms, while the other plays the role of a supportive friend. The friend will use mental health first aid skills to offer appropriate support.
**Stress Management**	Scavenger hunt		✓	✓			S	A 10-item game to practice what participants have learned through the asynchronous sessions. Each item is an interactive game which evaluates the knowledge about stress management lessons.
**Subjective Well-being**	Practice Mindfulness				✓		A	After receiving the training, participants would assess their emotional state. They would then practice mindfulness for a few minutes using a guided audio file and reassess their emotional state afterward.
	Practice Active-constructive Responding				✓	✓	A & S	In a simple simulated scenario, participants are presented with a set of described events and asked to select their preferred reactions from four provided options. Through this exercise, they become aware of their communication styles in various situations and understand the resulting consequences. Subsequently, during a synchronized session, they engage in role-play to practice the same activity.
**Stigma**	Arman		✓		✓		S	This activity aims to destigmatize mental illness. Students collaboratively create a character by building on the sentence, "Hi, my name is Arman, and I have been feeling blue lately…". At the end, they discuss whether they would want to be friends, colleagues, or neighbors with Arman, with the reminder that any of us could be in Arman’s position.

SBL, Scenario-Based Learning; SGD, Small Group Discussion; GBL, Game-based Learning; SRL, Self-Reflecting Learning; RP, Role-play; S, Synchronous; A, Asynchronous.

#### 2.1.5 Structure

In the scenario of this program, participants play their role as citizens trying to make a utopia for mental health, which the name of this program *Psycheutopia* is inspired by. Participants spend three weeks in this program; each week is specified for a topic (depression and suicidality, stress management, and well-being), while a longitudinal topic (stigma) goes on during the whole workshop. During each week, materials and contents are presented to participants as the asynchronous part of the flipped classroom. At the end of the week, they practice what they have learned under the guidance of their facilitators during a synchronous session in groups of six. This structure is specifically designed to improve participants’ skills as mentioned above. A detailed instruction for the program is provided in **Supplementary Material A**.

### 2.2 Implementation and evaluation

In this quasi-experimental pre-post test study, the didactic program *Psycheutopia* was implemented over a three-week online course in 2021. This study was conducted at Iran University of Medical Sciences in Tehran, Iran upon the approval of the Bioethics Committee of the Iran University of Medical Sciences.

#### 2.2.1 Participants

In this study, 43 students who volunteered to participate in this program were assessed for eligibility according to the following criteria: a) aged between 18 to 35 years, b) medical students, or alumni who graduated within the past six months, and c) able to participate in at least 80% of each synchronous session. Students with former experience in mental health, such as those who had participated in similar programs or mental health courses and those who had mental health-related professions, were excluded (n=11). Before the program’s initiation, the eligible participants provided informed consent to participate in this study, and they were assured that their personal information would be kept confidential.

#### 2.2.2 Intervention

Over three weeks, participants were divided into six groups, each supervised by a trained facilitator. These facilitators were students who had completed the pilot program and subsequently volunteered to join the team. Before the workshops, they underwent 10 hours of training, with their curriculum details outlined in **Supplementary Materials B**. Feedback on their performance was provided through fast-forward simulations, as described in **Supplementary Materials A**.

Each week focused on a specific theme: the first week addressed coping with depression and suicidal ideation, the second focused on stress management, and the third targeted improving overall well-being. Additionally, a longitudinal theme addressing stigma was integrated throughout the program.

Participants engaged in 90 minutes of asynchronous training weekly, followed by 270 minutes of interactive online sessions led by facilitators and trainers. Each topic included a comprehensive lesson plan with classroom activities and core and supplementary resources. To deliver the content, we used diverse media, including team-produced videos and podcasts. Influencers in the self-development field were also involved to enhance engagement. The program’s face and content validity were verified prior to implementation.

#### 2.2.3 Assessment and evaluation

Our primary outcome was improving mental health literacy by the end of the program. In other words, Participants should be able to understand how to obtain and maintain positive mental health, understand mental disorders, decrease their stigma related to mental disorders, and enhance their help-seeking efficacy.

Our secondary outcome was that in addition to improving subjective well-being, lay the groundwork for developing stress management skills and mental health first aid skills not just as a person as a medical professional but as a member of society.

Evaluating outcomes were through summative assessments using self-administered online questionnaires, including the Mental Health Literacy Scale (MHLS) (45), the Mental Health Promoting Knowledge (MHPK) (46), and the World Health Organization (WHO)-5 Well-being Index (WHO-5) (47) questionnaires at baseline, after the program and at four-month follow-up. These tools measured various domains of MHL, positive mental health literacy, and subjective well-being, The psychometric properties of the utilized questionnaires are detailed in **Supplementary Materials C**.

**Figure 3:**
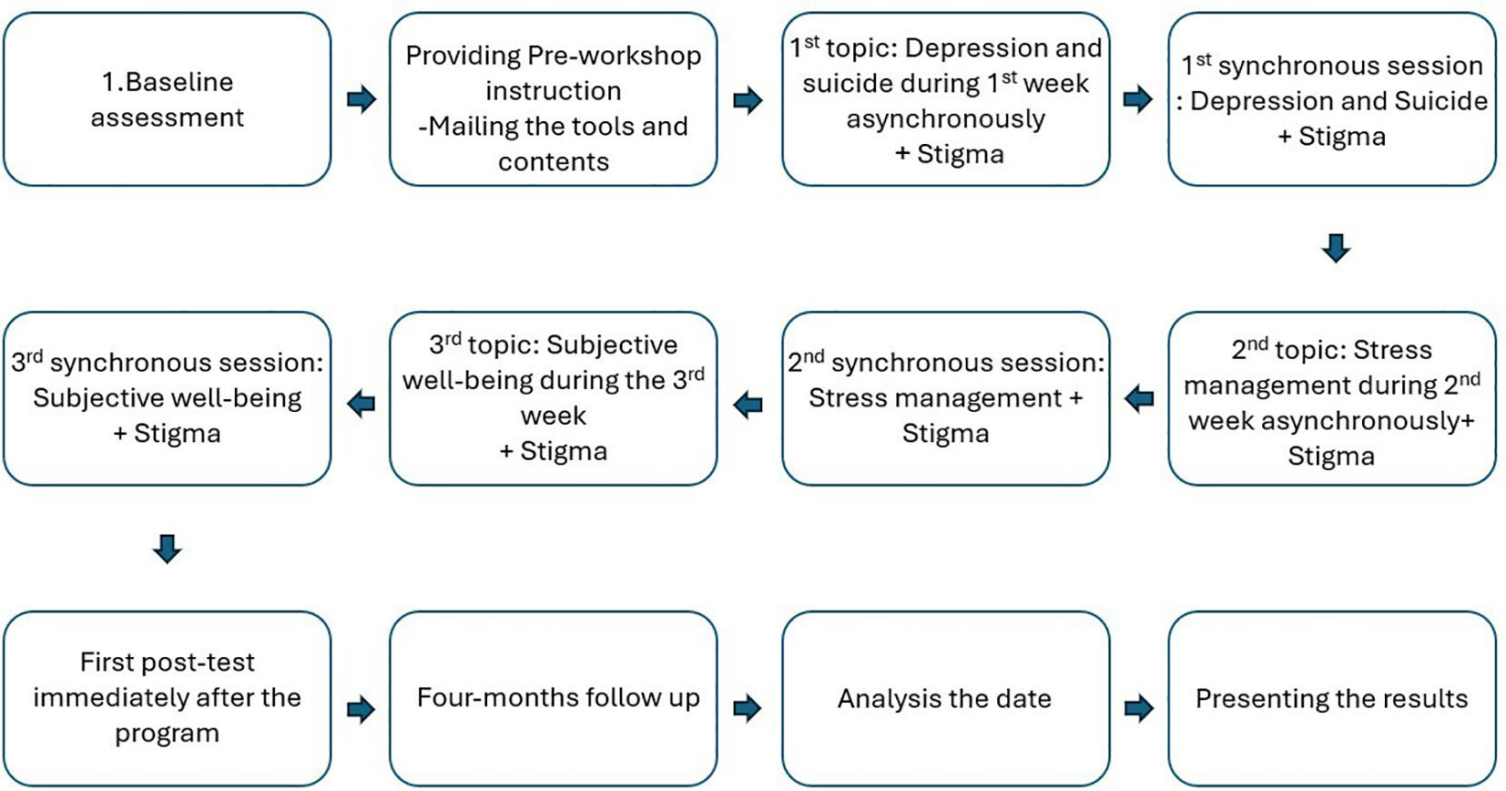
Temporal flow of intervention:implementation of the curriculum and evaluation of it.

Formative assessments also provided ongoing feedback during the program, involving self-reflective and peer assessments, with performance scored and ranked on a leaderboard (**Figure 3**).

#### 2.2.4 Program satisfaction assessment

Participants’ satisfaction with the program was evaluated at three-time points using a mixed-methods approach. A total of 17 quantitative questions were presented on a five-point Likert scale, addressing three domains:

1. Content satisfaction (four questions): Assessing the scientific quality and informativeness of the material.
2. Format satisfaction (four questions): Evaluating the presentation style and engagement.
3. Facilitator performance (nine questions): Measuring participants’ impressions of the facilitator’s skills and effectiveness.

In addition, seven open-ended qualitative questions were used to explore participants’ feedback in greater detail to identify general trends and consistency with quantitative findings.

### 2.3 Statistical method

The sample size was calculated using GPower software (version 3.1) for a two-sided paired t-test, with an expected effect size of 0.5, a significance level of 0.05, and a power of 80%. Adjustments for a potential dropout rate of 6% (2 participants) were also included, resulting in a planned recruitment of 32 participants to retain 30 effective participants.

The collected data were analyzed using SPSS 27.0. Numeric variables were reported as means with standard deviation (SD). Categorical variables were presented as percentages. The mean scores of the MHLS, MHPK, and SWB were analyzed according to a single-group design with repeated measurements analysis of variance (RM-ANOVA), and the Wilks’ Lambda test was used to test the normality of the data for the study variables. The graphs were created using GraphPad Prism version 9.1.1 for Mac, developed by GraphPad Software, San Diego, California, USA, available at www.graphpad.com. A two-sided significance level of 0.05 was used during the statistical testing and 95% confidence intervals (95% Cl) were calculated for the point estimates.

## 3 Results

**Figure 4:**
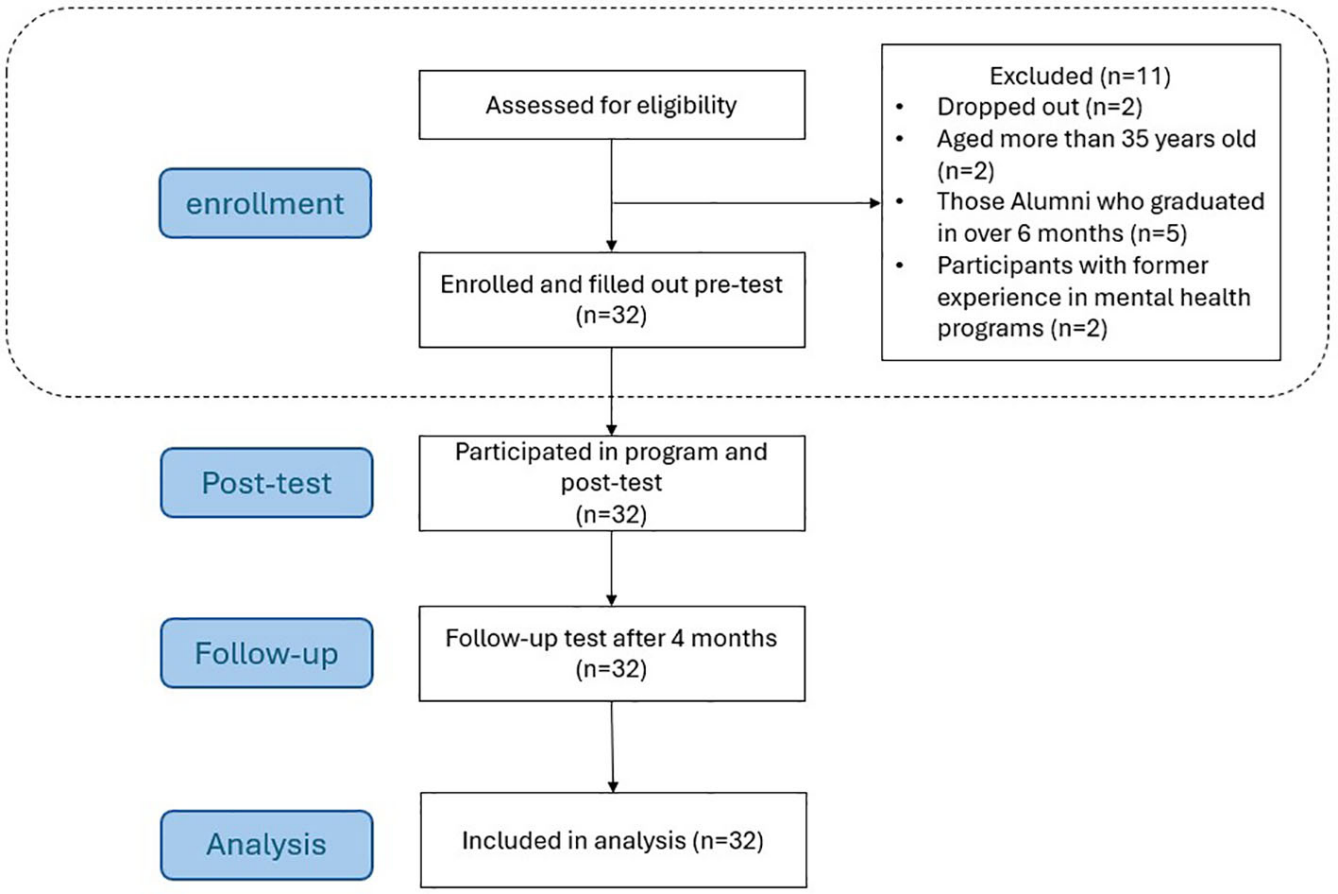
CONSORT flow diagram of participants selection.

A total of 43 medical students volunteered to enroll in this semi-experimental study. Thirty-two candidates, including 18 females and 14 males, fulfilled the eligibility criteria (**Figure 4**).

The mean age of the participants was 22.78 (4.54). At baseline, 53.1% of participants reported having a friend or family member with a psychiatric condition. Ninety percent of participants judged their mental health status as healthy or relatively healthy, and 68.1% had never been visited by a psychiatrist or a mental health professional (**Table 2**).

**Table 2:** Baseline characteristics of participants.

Characteristics		Mean (SD) N (%)
Age Mean (SD)		22.78 (4.54)
Gender n (%)	Male	14 (43.8)
	Female	18 (56.3)
Mental illness in acquaintances, n (%)	Yes	17 (53.1)
	No	15 (46.9)
Perceived mental health, n (%)	Completely healthy	0 (0)
	Relatively healthy	6 (18.7)
	Relatively ill	22 (68.7)
	Completely ill	3 (0.09)
	Undetermined	1 (0.03)
Mental health service use, n (%)	Yes	10 (31.3)
	No	22 (68.8)

A repeated measured ANOVA test revealed significant changes in MHLS (P-value<0.01(, MHPK (P-value=0.03), and SWB scores (P-value=0.02) over the four-month follow-up. The statistical results are summarized in **Table 3** (**Table 3**).

**Table 3:** MHLS, MHPK, and SWB scores before and after the intervention.

	Pre-test	Post-test	4-month follow-up	P-value
MHLS	77.1 (8.08)	80.7 (7.9)	82.63 (7.9)	0.004
MHPK	4.3 (0.5)	4.6 (0.4)	4.7 (0.2)	0.035
WHO-5 Well-being Index	51.6 (17.9)	60.4 (17.5)	58.0 (16.9)	0.027

Values are given as mean ± standard deviations of the mean. MHLS, Mental Health Literacy Scale; MHPK, Mental Health-Promoting Knowledge; WHO-5, World Health Organization (WHO)-5 well-being index (WHO-5); SWB, Subjective Well-being.

**Figure 5:**
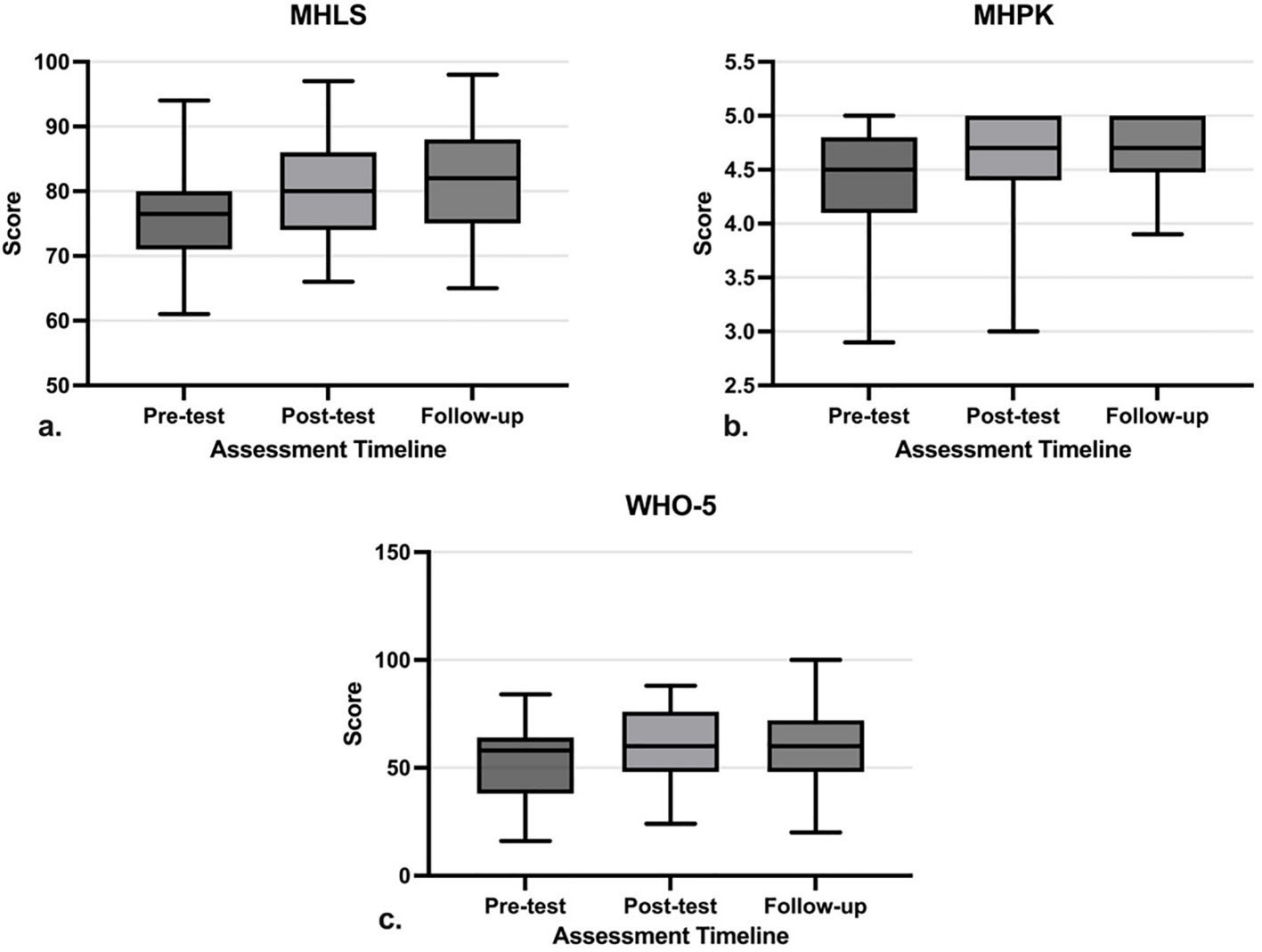
Trends of changes from pre-test to post-test and follow-up. The Whisker represents the range and the box demonstrates the 25th percentile (Q1) to the 75th percentile (Q3) with the median inside. **(a)** Mental Health Literacy Scale score, **(b)** Mental Health Promoting Knowledge score, **(c)** WHO-5 Wellbeing Index score.

As shown in **Figure 5**, the MHLS and MHPK scores significantly increased after the intervention (P-value= 0.02, P-value=0.03, respectively), and the changes were maintained during the four-month follow-up (P-value<0.01, P-value <0.01, respectively) in comparison with the baseline levels. Participants’ SWB scores increased by the end of the intervention (P-value=0.05, respectively); but decreased slightly after the workshop (**Figure 5c**) while remaining higher than baseline levels.

The quantitative analysis of participants’ satisfaction demonstrated consistently high ratings across all assessed domains. Among the domains, facilitator performance received the highest rating (M = 4.65, SD = 0.45), followed by content satisfaction (M = 4.43, SD = 0.46) and format satisfaction (M = 4.32, SD = 0.56).

## 4 Discussion

In this study, we designed and assessed the efficacy of *Psycheutopia*, a meticulously designed three-week online educational program aimed at enhancing the MHL among medical students. The program incorporated a range of interactive teaching methods and covered various aspects of mental health prevention and promotion. The results demonstrated a significant improvement in students’ MHL following the intervention, which was sustained at a four-month follow-up. Additionally, students’ well-being significantly improved both after the program and at the four-month follow-up. Our results demonstrated that participants with lower MHL showed substantial improvements following the intervention compared to those with higher MHL at the baseline.

The primary goal of this study was to enhance mental health literacy among medical students, with a focus on prevention and promotion. Mental health has been reframed into a spectrum ranging from complete health to illness, and it would be challenging to draw a line between different stages. Global mental health initiatives, such as the Lancet Commission, emphasize the importance of promoting mental health and reducing stigma as critical elements of sustainable development (48). To the best of our knowledge, only one educational program targeting medical specialties had a comprehensive approach to addressing the mental health continuum by far (49). Enhancing public awareness and encouraging help-seeking behaviors are particularly emphasized in Low- and Middle-Income Countries (LMICs), where targeted interventions like life skills education and stigma reduction are key strategies (50). While these interventions share common goals across populations, tailoring them to specific needs remains vital yet challenging.

This study demonstrated improvement in mental health literacy and positive mental health literacy after the program, which was sustained for four months. This result is consistent with other studies. Kurki et al. implemented a digital MHL program designed for medical students, focusing on life skills, stress management, and mindfulness. The program significantly improved mental health knowledge and reduced emotional symptoms, with these benefits persisting at a two-month follow-up. However, initial improvements in stress levels and help-seeking attitudes were not sustained, and stigma toward mental illness showed no significant change (49). In a peer-led program in university and secondary schools, Patalay et al. showed improvements in mental health literacy among school students, whereas a change in non-stigmatizing attitudes had the most significant effect size (51). Davies revealed that following a six-week e-learning mental health first aid program led to a decline in stigmatizing attitudes and improved helping intentions among medical students (52). A meta-analysis of 25 education interventions addressing MHL or stigma has shown these programs were associated with long-term improvements in MHL (53). It also emphasizes the importance of an educational condition to the outcome of the study. Consistent with our curriculum, this study suggests MHL is not limited to understanding symptoms and addressing other aspects of mental health literacy such as stigma, positive mental health literacy, and resilience is necessary and just relevant. On the other hand, the evidence around the long-term change in stigmatized attitudes following such interventions was less clear.

Another notable finding was the sustained improvement in well-being as the secondary outcome. Program components, such as stress management and practical skill-building, contributed directly to this outcome. Broader factors, including the program’s interactive nature, supportive environment, and the opportunity for meaningful engagement during the COVID-19 pandemic, may also have played a role. Some evidence suggests that interactive teaching methods can improve students’ well-being, regardless of the topic (54).

As stated in the Lancet Commission on Global Mental Health, the movement advocating for subjective well-being and happiness are one of the key indicators of a nation's development and it highlights the importance of promoting and protecting mental health for everyone, regardless of the presence of mental disorders (48). In our previous survey, we did not find a significant association between MHL and subjective well-being (9). Therefore, the improvement in well-being in this MHL educational program warrants further consideration.

The learning environment plays a crucial role in the success of such interventions. One of the primary challenges of mental health intervention in LMICs is the low compliance of the target population (55), which highlights the importance of using engaging and enjoyable teaching methods. Our teaching method was associated with a high level of satisfaction among participants while all participants completed the course of training. Participants highlighted the workshop’s engaging and informative content, the practicality of its delivery, and the facilitator’s expertise, collectively reflecting an overall very high level of satisfaction. Furthermore, the context-rich environment of *Psycheutopia* facilitated the translation of knowledge into practice. The relevance of the program to daily living situation, likely contributed to the sustained impact observed at the four-month follow-up. Consistent with our findings, previous studies have shown applying knowledge in real-world contexts, aligned with Bloom’s application level enhances student engagement, deepens understanding, and improves long-term retention (56). Moreover, incorporating student-centered elements into medical curricula can significantly reduce depressive symptoms (57).

The thoughtful design of the program and the novel use of various teaching methods to suit the audiences’ needs are other noteworthy features of this experience. Mohammadi identified five key factors that facilitate successful mental health interventions for adolescents: 1. Interactive learning environment, 2. Diverse and stimulating educational content, 3. trainers with different backgrounds, 3. Direct contact with people with mental disorders and 5. Utilizing technologies in education (37). These elements were integrated into the design of *Psycheutopia*, contributing to its effectiveness.

Sustainability and scalability are critical challenges in the design and implementation of such interventions (50). Similar to our experience, other successful online educational programs have found scenario-based or gamified teaching methods to be effective tools for creating a meaningful and engaging learning environment, for example, for nurses and adolescents (58, 59). Combining individual and team learning in an online environment may be a distinctive characteristic of Psycheutopia compared to similar interventions. In our program, learners exercise individually and then actively practice with other participants in online meetings.

Online interventions offer a cost-effective solution with broad accessibility. In a meta-analysis of 48 studies, the impact of internet-based mental health interventions on students has been evaluated as beneficial. These interventions have been found to enhance student's performance across various conditions (60). It is also recommended to reduce the role of specialists and facilitate community involvement in designing and implementing such interventions (48). Mental health program implemented by mental health professionals is associated with a significant cost, which poses a challenge to the feasibility and sustainability of such programs. Peer-delivered health promotion programs have shown efficacy in improving health literacy among students (51). Community-driven approaches further reduce reliance on specialists, addressing resource constraints in LMICs (48).

*Psycheutopia* exemplifies a community-based intervention created and implemented by medical students for their peers, building capacity under scientific supervision to make meaningful contributions. Students involved in the design and organization of the program not only contributed to its success but also benefited from learning and practicing the content themselves. Numerous interventions delivered by peers aim to improve young people's health, yet only a small number have been created and designed by young people themselves (61). Involving students in both the development and delivery of these programs has the potential to create more relevant and sustainable solutions (51). Additionally, evidence shows engaging students in the provision of a program is associated with their higher well-being and academic achievement, as well as long-term outcomes such as work success, personal development, and life-long learning (62). In several studies, the impact of peer-based mental health interventions on groups of students showed promising results (63–66), particularly in improving MHL (51). This concept is not limited to students, and the positive outcomes of Civil Engagement (CE) in the design and delivery of health services have been highlighted as an effective strategy to strengthen the health system (67, 68).

The primary limitation of this study is its semi-experimental design and lacking a control group. Although we compared measurements to students’ previous results using paired matches, the inclusion of a non-intervened group could have provided a clearer picture of the program’s efficacy.One limitation of our study is the selection of the target population, as participants were recruited on a voluntary basis. This self-selection process may introduce bias, as individuals who volunteered may differ in significant ways from the general population, such as being more motivated or health-conscious. Additionally, the majority of our participants were female, which may limit the generalizability of our findings to male populations. Future studies should aim for more diverse sampling methods to ensure a more representative distribution of participants across genders and other demographic factors. Additionally, while skill development was assessed through ongoing formative feedback, data on actual skill acquisition was not collected. Future studies should include skill development measures to complement knowledge-based outcomes. The subjective nature of well-being assessments is another limitation, and future research should explore different dimensions of well-being, such as affective and cognitive aspects, using more comprehensive measures. Additionally, mental health is influenced by various biological, developmental, and environmental factors. Thus, a holistic and systemic approach is essential to achieve meaningful improvements, with MHL enhancement as one component of broader mental health strategies.

## 5 Future implications

This study presents a feasible and replicable framework for integrating MHL programs into undergraduate curricula. Future research should evaluate the program’s applicability to the non-medical student population and assess its long-term efficacy.

## 6 Conclusions

*Psycheutopia* demonstrated the potential of an online educational intervention in increasing mental health literacy and improving the well-being of medical students. The results maintained after four months suggest that the achievements are persistent. The community-based nature of this experience highlights the importance of empowering students to train and support their peers. Key strategies for success included the use of evidence-based content, novel teaching methods, interactive learning, and a focus on making the experience enjoyable and relevant. These elements can be considered when designing future educational interventions for medical students.

## Data Availability

The raw data supporting the conclusions of this article will be made available by the authors, without undue reservation.
