# Supplementary material for: Psycheutopia: an innovative educational program to enhance mental health literacy among medical students
Source: Front Psychiatry. 2025 Mar 27;16:1538476. doi: 10.3389/fpsyt.2025.1538476 (PMC11982935; doi:10.3389/fpsyt.2025.1538476)
Supplement: Supplementary file 2 [file Presentation2.pdf]

Supplementary Materials B. The Curriculum for Facilitators' Training Program

Main Categories	Title of the Sessions	Details
Soft Skills	The role of a facilitator	❑ Understanding the dynamics of the group<br>❑ Developing Participation<br>❑ Understanding the barriers to participation and managing them
	Working with a team	❑ Effective communication skills<br>❑ Effective questioning skills<br>❑ Providing feedback effectively<br>❑ Conflict management<br>❑ Techniques for managing difficult situations
	Assessment skill	❑ Assessment and evaluation skills
	Facilitators' Toolbox	❑ Ice-breaking and energizer exercises<br>❑ Listening skills<br>❑ Time management
Professional Knowledge	Mental Health First Aids (MHFA)-Depression and suicide	❑ Reviewing the theoretical content, the educational objectives, and the activities of the program.<br>❑ How to assess the participants during each activity.<br>❑ How to access the program's designers while facing a difficulty.<br>❑ Understanding the program's conductor.
	Stress Management Skills	
	Subjective Well-being skills	
	Assessment guideline	❑ How to complete checklists and assess the participants' performance.
