# Supplementary material for: Psycheutopia: an innovative educational program to enhance mental health literacy among medical students
Source: Front Psychiatry. 2025 Mar 27;16:1538476. doi: 10.3389/fpsyt.2025.1538476 (PMC11982935; doi:10.3389/fpsyt.2025.1538476)
Supplement: Supplementary file 3 [file Presentation3.pdf]

Supplementary Materials C - the Psychometric Properties of the Questionnaires:

Mental Health Literacy Scale (MHLS):

MHLS is a questionnaire that measures the main domains of mental health literacy in five subscales including Knowledge about mental illness; Knowledge about confidentiality of information; Access to information; Knowledge about mental illness treatment; and stigma(1). The Persian version consists of twenty-three items and is valid and reliable with Cronbach's alpha of 0.873 (2) and the total score ranges from 23 to 106. Higher scores indicate better mental health literacy.

Mental Health-Promoting Knowledge (MHPK):

MHPK is a 10-item questionnaire that evaluates positive mental health literacy which is a person's awareness of how to achieve and maintain appropriate mental health(3). Each item is rated on a Likert scale (e.g., 1 = strongly disagree, 4 = strongly agree), resulting in a total score range of 10 to 40. Higher scores reflect greater knowledge about mental health promotion. The Persian version is valid and reliable with Cronbach's alpha of 0.81 (4).

World Health Organization (WHO)-5 Well-being Index:

The WHO-5 questionnaire consists of five questions and evaluates individuals' subjective well-being. It contains five questions, and each item is scored on a Likert scale from 0 to 5. The raw scores are summed (0-25) and then transformed to a scale of 0 to 100. A transformed score of ≤50 is commonly used as a cutoff to indicate potential depression and may warrant further assessment. Higher scores indicate better well-being. The Persian version is a valid and reliable questionnaire with Cronbach's alpha of 0.89 to 0.91 (5-7).

References:

1. O’Connor M, Casey L. The Mental Health Literacy Scale (MHLS): A new scale-based measure of mental health literacy. Psychiatry research. 2015;229(1-2):511-6.
2. Ghaedamini Harouni G, Sajjadi H, Forouzan AS, Ahmadi S, Ghafari M, Vameghi M. Validation of the Persian version of the mental health literacy scale in Iran. Asia-Pacific Psychiatry. 2022;14(1):e12447.
3. Bjørnsen HN, Eilertsen MEB, Ringdal R, Espnes GA, Moksnes UK. Positive mental health literacy: development and validation of a measure among Norwegian adolescents. BMC public health. 2017;17:1-10.
4. Mahmoodi MH, Ahmadzad-Asl M, Rasoulian M. Mental health positive knowledge questionnaire: translation and cultural adaptation and psychometric properties. Social Welfare Quarterly. 2021;21(82):245-73.
5. Topp CW, Østergaard SD, Søndergaard S, Bech P. The WHO-5 Well-Being Index: a systematic review of the literature. Psychotherapy and psychosomatics. 2015;84(3):167-76.
6. Dehshiri G, Mousavi S. An investigation into psychometric properties of persian version of World Health Organization Five Well-Being Index. Journal of clinical psychology. 2016;8(2):67-75.
7. Dadfar M, Momeni Safarabad N, Asgharnejad Farid AA, Nemati Shirzy M, Ghazie pour Abarghouie F. Reliability, validity, and factorial structure of the World Health Organization-5 Well-Being Index (WHO-5) in Iranian psychiatric outpatients. Trends in psychiatry and psychotherapy. 2018;40:79-84.
